# Identification of Ras suppressor-1 (RSU-1) as a potential breast cancer metastasis biomarker using a three-dimensional *in vitro* approach

**DOI:** 10.18632/oncotarget.16062

**Published:** 2017-03-09

**Authors:** Vasiliki Gkretsi, Andreas Stylianou, Maria Louca, Triantafyllos Stylianopoulos

**Affiliations:** ^1^ Cancer Biophysics Laboratory, Department of Mechanical and Manufacturing Engineering, University of Cyprus, Nicosia, Cyprus

**Keywords:** Ras suppressor-1, extracellular matrix stiffness, invasion, tumor spheroids, atomic force microscopy

## Abstract

Breast cancer (BC) is the most common malignant disease in women, with most patients dying from metastasis to distant organs, making discovery of novel metastasis biomarkers and therapeutic targets imperative. Extracellular matrix (ECM)-related adhesion proteins as well as tumor matrix stiffness are important determinants for metastasis. As traditional two-dimensional culture does not take into account ECM stiffness, we employed 3-dimensional collagen I gels of increasing concentration and stiffness to embed BC cells of different invasiveness (MCF-7, MDA-MB-231 and MDA-MB-231-LM2) or tumor spheroids. We tested the expression of cell-ECM adhesion proteins and found that Ras Suppressor-1 (RSU-1) is significantly upregulated in increased stiffness conditions. Interestingly, RSU-1 siRNA-mediated silencing inhibited Urokinase Plasminogen Activator, and metalloproteinase-13, whereas tumor spheroids formed from RSU-1-depleted cells lost their invasive capacity in all cell lines and stiffness conditions. Kaplan-Meier survival plot analysis corroborated our findings showing that high RSU-1 expression is associated with poor prognosis for distant metastasis-free and remission-free survival in BC patients. Taken together, our results indicate the important role of RSU-1 in BC metastasis and set the foundations for its validation as potential BC metastasis marker.

## INTRODUCTION

Breast cancer (BC) metastasis is known to account for most cancer-associated deaths in women [1]. Although significant research has been conducted in the area of cancer metastasis, to date there is no available marker to predict metastasis nor is there any way to inhibit it once it has occurred. Evidently, there are still open questions regarding the exact mechanism involved, while it has been established that the extracellular matrix (ECM) and ECM-related adhesion proteins are key molecules in the process of metastasis. Indeed cell-ECM adhesion proteins can be greatly disrupted in cancer allowing tumor cells to detach from the original tumor mass [2–5], adhere to the endothelium and adjacent tissues, and invade through them [6]. Moreover, connection of cell-ECM adhesions to the actin cytoskeleton enables cells to respond to external stimuli by modulating their shape or by activating proper migration patterns.

Ras Suppressor-1 (RSU-1) was recently shown to localize to cell-ECM adhesion sites through its interaction with Particularly Interesting New C*ysteine-Histidine* rich protein (PINCH-1) [7], which in turn binds to Integrin-Linked Kinase (ILK), and alpha-parvin (PARVA) forming a stable ternary protein complex that promotes cell survival [8–10]. Although, RSU-1 was originally identified as suppressor of Ras-dependent oncogenic transformation [11], little is known regarding its expression and role in cancer. From the studies currently published on RSU-1 and cancer, there is consensus on the fact that RSU-1 has anti-tumorigenic effects suppressing cancer cell growth [11–14]. Regarding its expression in various precancerous or cancer tissues though, results are limited and sometimes contradictory. A study in familial adenomatous polyposis involving a small number of samples showed a reduction in RSU-1 protein expression in polyposis samples compared to normal colonic mucosa [15] while another study showed RSU-1 mRNA expression to be dramatically up-regulated in metastatic colon cancer samples compared to healthy controls as well as compared to primary colon cancer samples [16]. Furthermore, a somatic copy number variation (CNV) analysis in hepatocellular carcinoma samples showed that the *Rsu-1* gene exhibited a high frequency of CNVs with 7 deletions and 3 amplifications [17] indicating that *Rsu-1* is frequently deleted in human liver cancer. Moreover, it was recently shown that RSU-1 expression is significantly elevated both at the mRNA and protein level in BC samples compared to respective adjacent normal tissue with the increase being more obvious in metastatic samples compared to non-metastatic [18]. Consistent with this finding, RSU-1 was demonstrated to be significantly upregulated in the aggressive MDA-MB-231 breast cancer cells compared to less aggressive MCF-7 cells [18], as well as in the aggressive HepG2 hepatocellular carcinoma cells compared to the less invasive PLC/PRF/5 (Alexander) hepatoma cells [19]. Interestingly, an alternatively-spliced variant of *rsu-1* was identified in 30% of high grade gliomas and 2/3 of oligodendrogliomas but not in other brain, bladder, colon tumors of normal tissue [20] while rare RSU-1 deletion were also identified in three cancer types from the Cancer Genome Atlas [21]. Hence, RSU-1 seems to have the potential of being both promising and clinically relevant novel marker and therapeutic target of cancer cell metastasis. Apart from the involvement of cell-ECM adhesion proteins, it has also been shown that mechanical cues can promote cancer metastasis [22, 23]. In fact, cancer tissues often contain a larger amount of ECM proteins than normal tissues and thus, are typically stiffer, expressed with a larger value of Young's modulus. Tumor stiffening is the only mechanical aspect that patients and clinicians can feel as in many cases tumors become stiffer compared to the surrounding tissue. Because of their increased ECM stiffness, cancer tissues restrict more the movement of cancer cells, exerting larger mechanical compressive forces on them. Thus, mechanical compression can, not only reduce cancer cell proliferation and induce apoptosis but it can also increase the invasive and metastatic potential of cancer cells [6, 22–30].

In the current study, we set out to investigate the role of cell-ECM adhesion proteins in relation to matrix stiffness with regard to cell invasion. Traditional two-dimensional (2D) monolayer cultures could not be used, as they cannot take into account the ECM stiffness of the tumor microenvironment [31]. Thus, in order to better approximate the real tumor setting *in vitro*, we used two approaches: a) we cultured BC cells in three dimensional (3D) collagen I gels of increasing concentration and stiffness [31] studying the expression of cell-ECM adhesion proteins including but not limited to RSU-1, and b) we generated cancer cell spheroids, embedded in 3D collagen gels of increasing stiffness and monitored their invasive capacity in the presence or absence of RSU-1.

## RESULTS

### Defining the 3D culture system of collagen I gels in terms of stiffness

**Figure 1:**
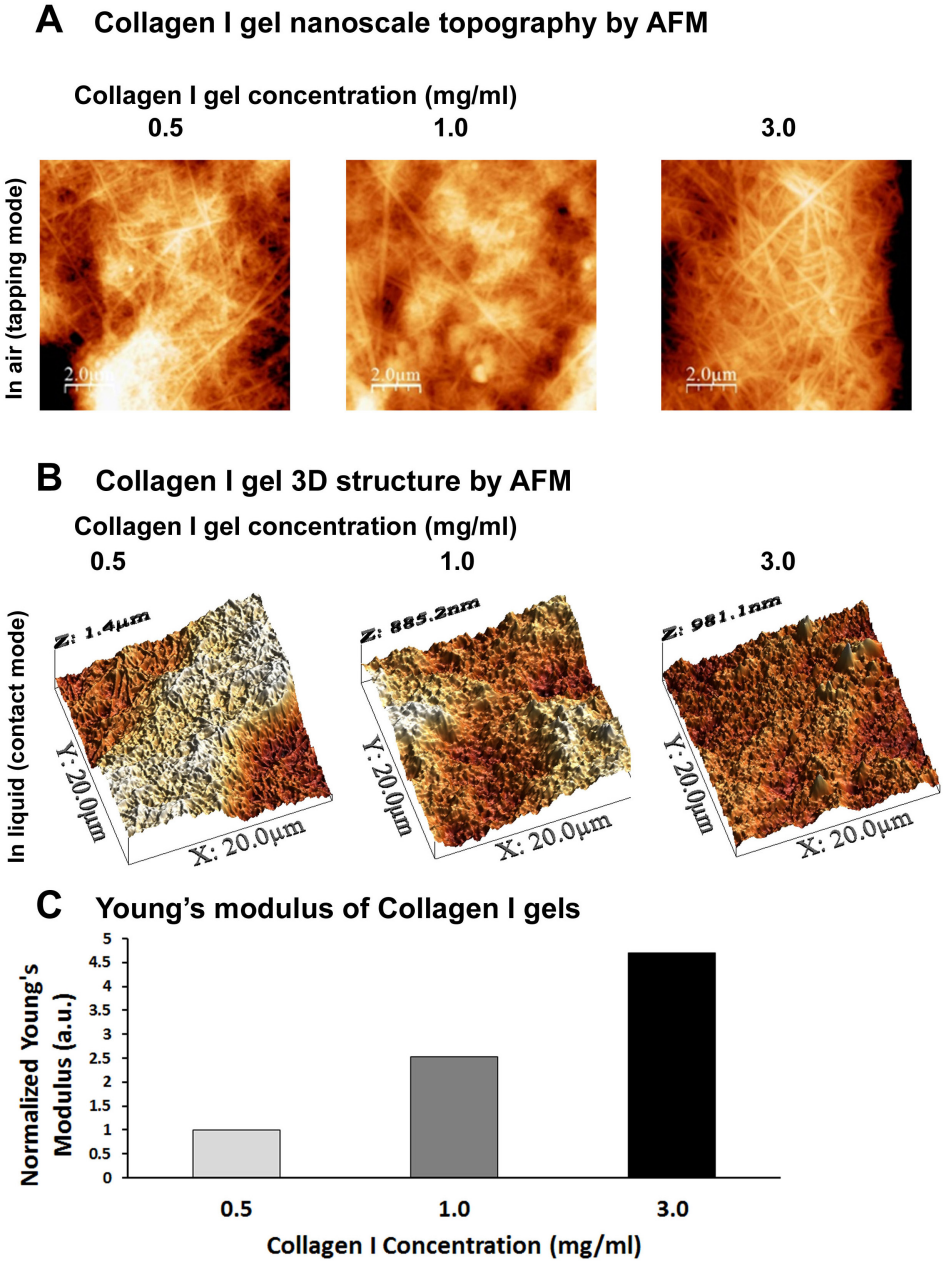
Defining the 3D culture system of Collagen I gels by AFM **(A)** AFM Topography images of the nanosurface of collagen I gels (tapping mode in air, with Cypher ES, Asylum Research AFM system). **(B-C)** AFM 3D topography images in PBS and diagrammatic representation of normalized Young's modulus of the gels (contact mode and force spectroscopy under liquid, with PicoPlus, Agilent AFM System).

We first set out to establish a 3D culture system that would allow cancer cells to grow in 3D and at the same time allow us to modulate the stiffness of the surrounding matrix, in an attempt to better approximate real tumor setting and the cell-ECM interactions in place. To that regard, we generated collagen I gels containing 0.5, 1.0 or 3.0 mg/ml collagen I. In order to characterize the generated gels in terms of structure and stiffness, we employed AFM, which can be used for imaging and characterization of the mechanical properties of collagen samples without destroying the fibrillar structure of collagen [32, 33]. As shown in Figure 1A, collagen gels consisted of fibers with 3D random orientations, confirming that the formed gels mimic collagen-rich tissues. Furthermore, the density of the fibers in the gels increased with collagen concentration. In order to characterize the stiffness of the gels, we performed AFM analysis under liquid conditions. Figure 1B depicts 3D structural images of collagen gels taken in PBS that were used to generate the stiffness graph in Figure 1C (normalized Young's modulus values). As expected according to previous studies [34, 35], the stiffness measured by AFM increases with increasing collagen concentration. In particular, we measured an increase in stiffness of about 1.5 and 3.7 times (compared to the 0.5mg/ml condition), when the concentration was increased from 0.5 to 1.0 and 3.0 mg/ml, respectively.

### BC cells grown in 3D collagen gels of increasing stiffness

**Figure 2:**
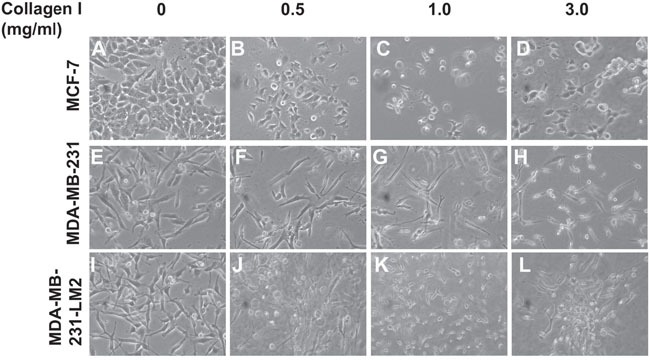
BC cells grown in 3D collagen gels in conditions of increasing matrix stiffness **(A-D)** Morphology of MCF-7 cells grown in 2D culture **(A)**, or embedded in Collagen gels of 0.5mg/ml **(B)**, 1.0 mg/ml **(C)** and 3.0 mg/ml **(D)**. **(E-H)** Morphology of MDA-MB-231 cells grown in 2D culture **(E)**, or embedded in Collagen gels of 0.5 mg/ml **(F)**, 1.0 mg/ml **(G)** and 3.0 mg/ml **(H)**. **(I-L)** Morphology of MDA-MB-231-LM2 cells grown in 2D culture **(I)**, or embedded in Collagen gels of 0.5mg/ml **(J)**, 1.0 mg/ml **(K)** and 3.0 mg/ml **(L)**. Pictures were taken using a Nicon Eclipse TS100 optical microscope equipped with digital camera.

Subsequently, we proceeded with cell culturing by embedding BC cells in the gels, according to previously published protocols [36, 37], allowing them to grow for 3 days. In all our experiments we used three BC cell lines of different metastatic potential; the non-invasive MCF-7 cells, the highly invasive MDA-MB-231 cells and the also highly invasive MDA-MB-231-LM2 cells, which were derived from MDA-MB-231 cells and were selected for their ability to metastasize to lung tissue *in vivo* [38]. As shown in Figure 2, MCF-7 (Figure 2A-2D), MDA-MB-231 (Figure 2E-2H) and MDA-MB-231-LM2 cells (Figure 2I-2L) were indeed embedded in the gels growing at different levels in all three dimensions within the 3D collagen matrix. The different levels of focus, seen in the pictures, involving cells grown in the gels confirm our observations (Figure 2B-2D, Figure 2F-2H, Figure 2J-2L).

### BC cell spheroids’ invasion within collagen gels is increased at medium stiffness and decreased at high stiffness conditions

**Figure 3:**
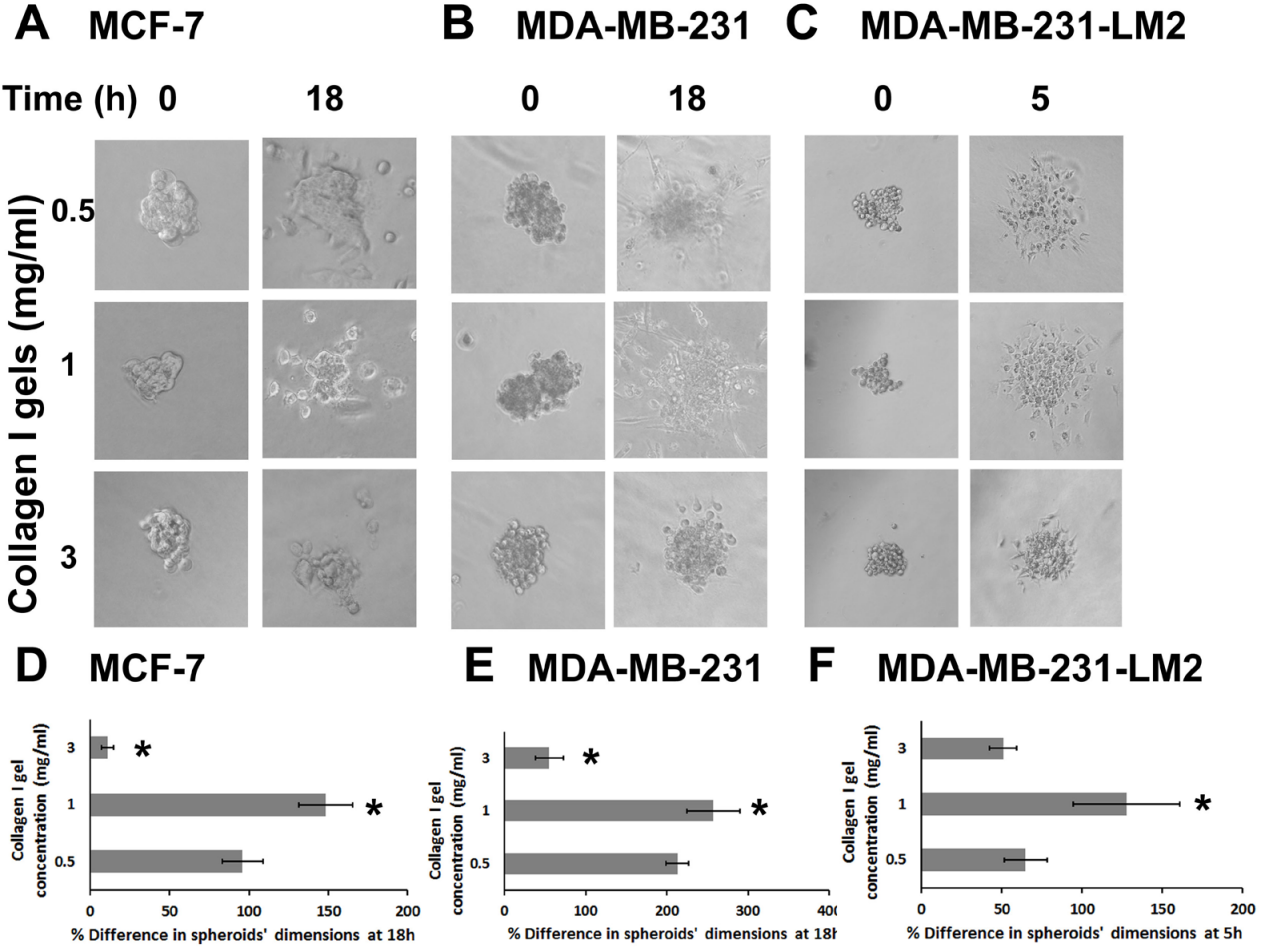
Cancer cell spheroids invasion through collagen gels is increased at medium stiffness and decreased at high stiffness conditions **(A-C)** MCF-7, MDA-MB-231 and MDA-MB-231-LM2 cell spheroids embedded in collagen gels of 0.5, 1.0 or 3.0 mg/ml at time zero and at 18h, 18h and 5h post implantation, respectively. **(D-F)** Percentage (%) change in MCF-7, MDA-MB-231 and MDA-MB-231-LM2 spheroids’ dimensions (average of major and minor axis) within 18h, 18h and 5h post implantation, respectively. At least 8 spheroids were analyzed per condition and 3 independent experiments were performed. Asterisks indicate statistically significant changes (p value < 0.05) compared to the condition of 0.5 mg/ml.

We then sought to find out how the metastatic potential of BC cells is affected by ECM stiffness. To better control invasion routes of cells and better recapitulate a breast tumor *in vitro* we generated BC cell spheroids using the hanging drop method [30, 39, 40], and implanted them in culture wells containing 0.5, 1.0 or 3.0 mg/ml collagen gels, considering the time of implantation as time zero. Pictures of the spheroids were taken at time zero, as well as at 2, 5 and 18h, and the spheroid size (mean of major and minor axis length) was compared to the initial size. As shown in Figure 3, in all three cell lines tested, BC cells invaded through 0.5 and 1.0 mg/ml collagen and the size of the spheroid was increased over time, although this was not the case for spheroids implanted in the 3.0 mg/ml collagen gel where the spheroids’ size showed significant decrease (Figure 3D-3F). This indicates that the relationship between cells’ invasive capacity and ECM stiffness is biphasic, with an optimum cell invasion occurring at intermediate stiffness conditions (i.e. 1.0 mg/ml collagen). It is worth noting that MCF-7 cell spheroids needed at least 18h to invade through surrounding matrix, while MDA-MB-231 spheroids reached double the size of MCF-7 spheroids in the same time period (compare Figure 3D and 3E). As expected, the highly metastatic MDA-MB-231-LM2 cell spheroids reached similar size at only 5h (Figure 3F).

### Effect of 3D culture on cell-ECM adhesion gene expression

Following, we set out to investigate how proteins involved in cell-ECM interactions at cell-ECM adhesion sites are affected by 3D culture, as these are the first to receive the mechanical signals from the ECM and transmit them further to the interior of the cell enabling it to survive, reorganize its cytoskeleton and regulate its homeostasis. Hence, we tested the expression of cell-ECM adhesion components ILK, PARVA, migfilin, Vasodilator Stimulated Phosphoprotein (VASP), PINCH-1, and RSU-1. As shown in Supplementary Figure 1, ILK, PARVA, VASP and Migfilin mRNA and protein expression was not following a consistent trend in relation to increased collagen stiffness among the three cell lines tested. PINCH-1 protein expression also did not follow a specific pattern, although PINCH-1 mRNA expression was significantly reduced in increasing stiffness conditions in MCF-7 and MDA-MB-231 cells, but not in MDA-MB-231-LM2 cells (Supplementary Figure 2).

**Figure 4:**
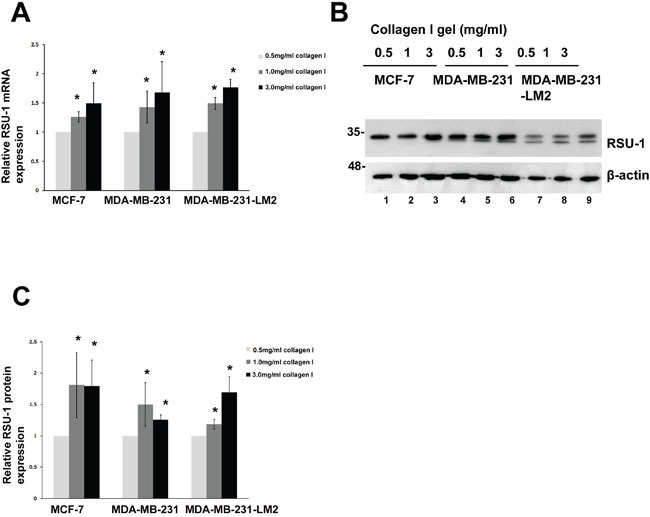
RSU-1 is upregulated in increased stiffness conditions in MCF-7, MDA-MB-231 and MDA-MB-231-LM2 cells **(A)** Relative RSU-1 mRNA expression in MCF-7, MDA-MB-231 and MDA-MB-231-LM2 cells cultured in collagen gels of 0.5, 1.0 and 3.0 mg/ml. Seven (7) independent Real Time PCR experiments were performed, and data were analyzed using the ΔΔCt method and having 0.5mg/ml collagen gel as a calibrator. **(B)** Representative western blot showing RSU-1 protein expression in all stiffness conditions in all three BC cell lines. B-actin was utilized as loading control. **(C)** Graph representing quantification of RSU-1 protein expression normalized to the β-actin loading control using NIH Image J software. The mean intensity of RSU-1 protein bands from 7 different immunoblots was used for the quantification. Asterisks indicate statistically significant changes (p-value<0.05).

Interestingly however, RSU-1 was the only one, among cell-ECM adhesion components tested, to show a consistent differential response in expression in relation to increased collagen stiffness in all three BC cell lines. More specifically, RSU-1 expression was significantly elevated in cells grown in 1.0 or 3.0 mg/ml collagen gels, compared to cells grown in 0.5 mg/ml collagen gels, both at the mRNA (Figure 4A) and protein level (Figure 4B and 4C), indicating a potential connection between RSU-1 and ECM-originating mechanical cues.

### RSU-1 is upregulated in more aggressive BC cell lines

**Figure 5:**
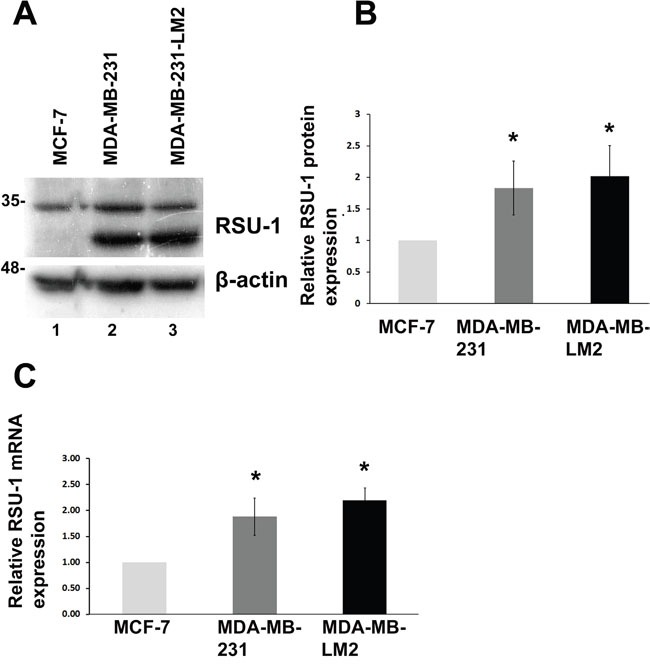
RSU-1 mRNA and protein expression is elevated in more aggressive BC cells compared to less aggressive **(A)** Representative western blot showing RSU-1 protein expression in MCF-7, MDA-MB-231 and MDA-MB-231-LM2 cells. B-actin was utilized as loading control. **(B)** Graph representing quantification of RSU-1 protein expression normalized to the β-actin loading control using NIH Image J software. The mean intensity of RSU-1 protein bands from 6 different immunoblots was used for the quantification. **(C)** Relative RSU-1 mRNA expression in MCF-7, MDA-MB-231 and MDA-MB-231-LM2 cells cultured in traditional 2D culture conditions. Four independent Real Time PCR experiments were performed, and data were analyzed using the ΔΔCt method and having MCF-7 cell sample as calibrator. Asterisks indicate statistically significant changes (p-value<0.05).

Taking into consideration the fact that RSU-1 is also linked to cancer as it was originally identified as suppressor of Ras-induced transformation [11] and it was recently associated with metastasis in BC patients [18], we wondered what would be its involvement in BC progression and metastasis and how it is affected by matrix stiffness. Thus, we first tested RSU-1 expression in 2D culture and found that RSU-1 is upregulated in the highly invasive MDA-MB-231 and MDA-MB-231-LM2 cells compared to the less aggressive MCF-7 cells both at the protein (Figure 5A and 5B) and mRNA (Figure 5C) level.

### RSU-1 is efficiently silenced in all three BC cell lines in 2D culture and its silencing leads to reduced UPA, and MMP-13 mRNA expression

**Figure 6:**
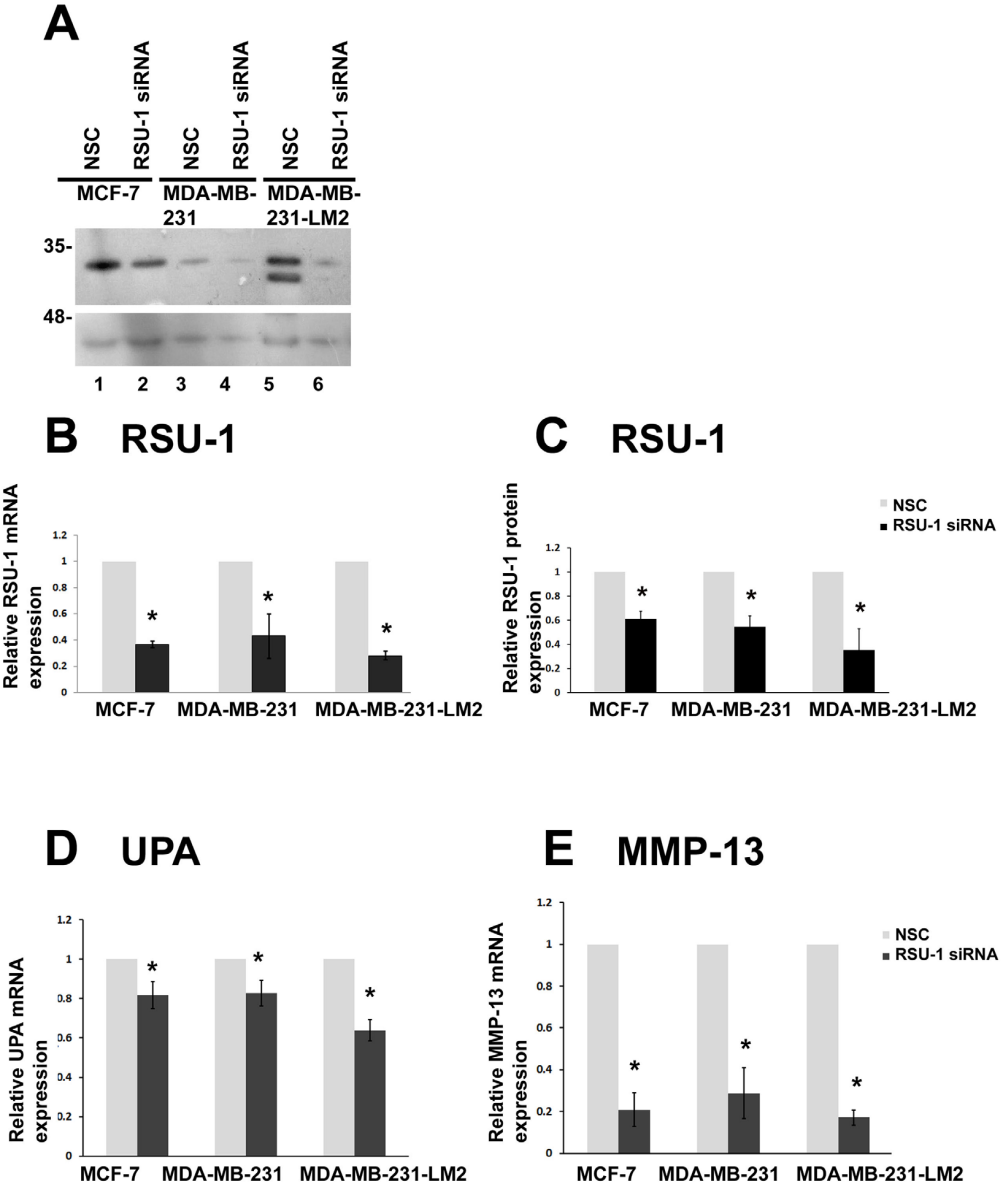
RSU-1 is effectively silenced in 2D conditions and its silencing leads to downregulation of UPA, and MMP-13 in all three BC cell lines **(A)** Representative western blot showing RSU-1 protein expression in all MCF-7, MDA-MB-231 and MDA-MB-231-LM2 cells treated with NSC or RSU-1 siRNA for at least 48h. B-actin was utilized as loading control. **(B)** Relative mRNA expression of RSU-1 in MCF-7, MDA-MB-231 and MDA-MB-231-LM2 cells cultured in traditional 2D culture conditions and treated with NSC or RSU-1 siRNA for at least 48h. Seven independent Real Time PCR experiments were performed, and data were analyzed using the ΔΔCt method and having NSC-treated cells as calibrators. **(C)** Graph representing quantification of RSU-1 protein expression normalized to the β-actin loading control using NIH Image J software. The mean intensity of RSU-1 protein bands from 4 different immunoblots was used for the quantification. **(D-E**) Relative mRNA expression of UPA **(D)**, and MMP-13 **(E)** in MCF-7, MDA-MB-231 and MDA-MB-231-LM2 cells cultured in traditional 2D culture conditions and treated with NSC or RSU-1 siRNA for at least 48h. At least 3 independent Real Time PCR experiments were performed, and data were analyzed using the ΔΔCt method and having NSC-treated cells as calibrators. Asterisks indicate statistically significant changes (p-value<0.05).

In order to decipher the role of RSU-1 in BC cells in relation to metastasis, we first performed siRNA-mediated gene silencing in all three BC cell lines cultured in 2D. RSU-1 was significantly and effectively silenced both at the protein (Figure 6A and 6C) and mRNA (Figure 6B) level compared to cells being transfected with non-specific control (NSC) siRNA. A recent study has demonstrated that RSU-1 elimination from aggressive hepatocellular carcinoma cells inhibits cell invasion [19], although the molecular mechanism involved was not provided. Thus, we wanted to test whether RSU-1 silencing affects key molecules involved in matrix degradation. To that regard and following RSU-1 silencing, we tested the mRNA expression of Urokinase Plasminogen Activator (UPA), a known protease involved in cancer progression and metastasis, as well as that of metalloproteinase-13 (MMP-13) known to be involved in collagen I degradation. We found that both UPA (Figure 6D), and MMP-13 (Figure 6E) were significantly downregulated following RSU-1 silencing compared to NSC-treatment in all three BC cell lines, indicating a strong inhibitory effect of RSU-1 silencing on BC cell invasive potential.

### RSU-1 is efficiently silenced in all three BC cell lines in 3D collagen gels of increasing stiffness and its elimination leads to reduced UPA, and MMP-13 mRNA expression

**Figure 7:**
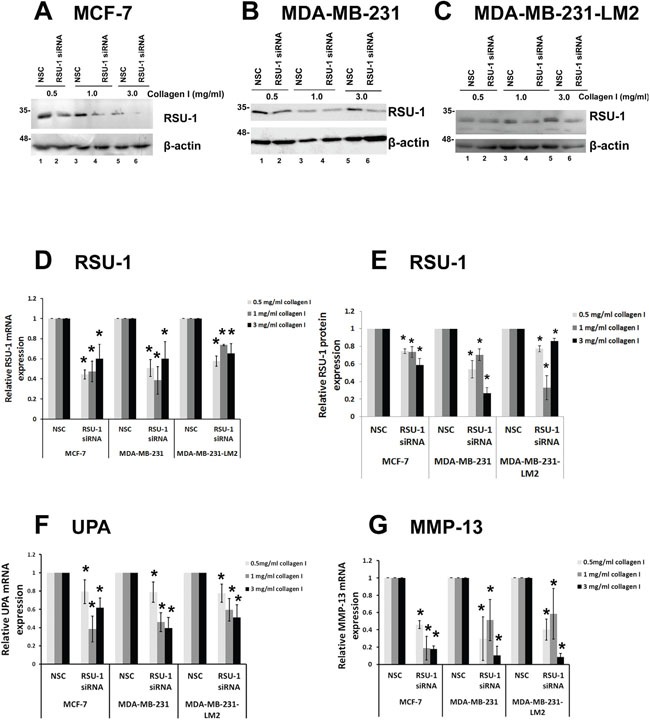
RSU-1 is effectively silenced in 3D conditions and its elimination downregulates UPA, and MMP-13 in all three BC cell lines **(A-C)** Representative western blots showing RSU-1 protein expression in MCF-7 (A), MDA-MB-231 (B) and MDA-MB-231-LM2 (C) cells treated with NSC or RSU-1 siRNA for 72h being also embedded in collagen matrix of 0.5, 1.0 or 3.0 mg/ml. B-actin was utilized as loading control. **(D**) Relative mRNA expression of RSU-1 (D), in MCF-7, MDA-MB-231 and MDA-MB-231-LM2 cells treated with NSC or RSU-1 siRNA for 72h and being also embedded in collagen matrix of 0.5, 1.0 or 3.0 mg/ml. **(E)** Graph representing quantification of RSU-1 protein expression normalized to the β-actin loading control using NIH Image J software. The mean intensity of RSU-1 protein bands from three different immunoblots per cell line were used for the quantification. Asterisks indicate statistically significant changes (p-value<0.05). **(F-G)** UPA (F) and MMP-13 (G) relative mRNA expression in MCF-7, MDA-MB-231 and MDA-MB-231-LM2 cells treated with NSC or RSU-1 siRNA for 72h and being also embedded in collagen matrix of 0.5, 1.0 or 3.0 mg/ml. At least three independent Real Time PCR experiments were performed, and data were analyzed using the ΔΔCt method and having NSC-treated cells as calibrators in each stiffness condition. Asterisks indicate statistically significant changes (p-value <0.05).

Next, we performed siRNA-mediated RSU-1 silencing in all three BC cell lines prior to embedding them in 3D collagen gels, where they were allowed to grow for two additional days. Morphology of the cells grown in 3D conditions of increased stiffness following RSU-1 siRNA was not significantly altered by RSU-1 elimination (Supplementary Figure 3). RSU-1 was effectively silenced in all three cell lines treated with RSU-1 siRNA, and in all three collagen stiffness conditions compared to the respective NSC-treated cells both at the protein (Figure 7A-7C, 7E) and mRNA level (Figure 7D).

To test whether RSU-1 silencing has the same effect in 3D collagen cultures as it has in 2D, we checked UPA and MMP-13 mRNA expression in all three cell lines and all three stiffness conditions after RSU-1 silencing. In accordance with findings from the 2D culture experiments (Figure 6), RSU-1 elimination also significantly inhibited the mRNA expression of UPA (Figure 7F) and MMP-13 (Figure 7G).

### RSU-1 silencing in cancer cell spheroids implanted in 3D collagen gels leads to reduced cell invasion in a stiffness-independent manner

**Figure 8:**
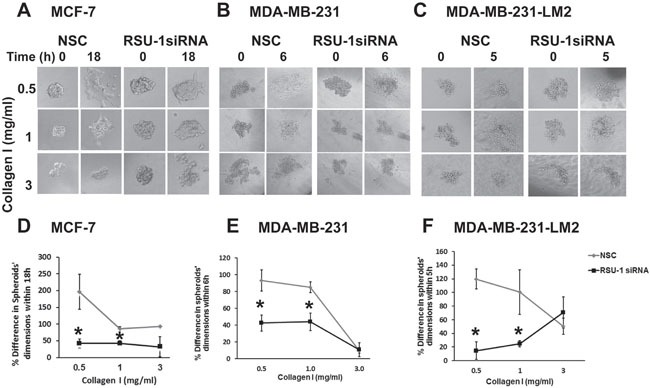
RSU-1 silencing in BC cell spheroids implanted in 3D collagen gels leads to reduced cell invasion in a stiffness-independent manner **(A-C)** MCF-7, MDA-MB-231 and MDA-MB-231-LM2 cells treated with NSC or RSU-1 siRNA were used to generate BC cell spheroids that were subsequently embedded in collagen gels of 0.5, 1.0 or 3.0 mg/ml at time zero and left to grow for 18h, 6h and 5h post implantation, respectively. **(D-F)** Percentage (%) change in MCF-7, MDA-MB-231 and MDA-MB-231-LM2 spheroids’ dimensions (average of major and minor axis) within the specified times post implantation. At least 8 spheroids were analyzed per condition and 3 independent experiments were performed. Asterisks indicate statistically significant changes (p-value < 0.05).

Having already shown that RSU-1 depletion leads to reduction in the mRNA expression of matrix degrading proteases UPA, and MMP-13 both in 2D and 3D cell culture conditions, we proceeded further to test the actual effect on invasiveness of BC cell spheroids. Thus, MCF-7, MDA-MB-231 and MDA-MB-231-LM2 cells were subjected to RSU-1 siRNA transfection and one day later were used to generate cancer cell spheroids that were subsequently implanted in collagen gels of increasing stiffness 24h later (48h post-siRNA transfection). Pictures were taken at the time of implantation (time zero) and at the optimum time for each cell line which was determined following pilot experiments and was 18h for MCF-7 cells, 6h for MDA-MB-231 cells and 5h for MDA-MB-231-LM2 cells. RSU-1 silencing in all three BC cell lines resulted in significant inhibition of BC cell spheroid invasion in 0.5, and 1.0 mg/ml collagen matrices but had no effect on spheroids embedded in 3.0 mg/ml collagen gels (Figure 8). In this concentration invasiveness is already low, even without silencing (Figure 3). Importantly, the effect of inhibition was more dramatic in the more aggressive and highly metastatic MDA-MB-231-LM2 cells (Figure 8C and 8F). Thus, our findings indicate that RSU-1 depletion inhibits BC cell invasion without any apparent dependence on matrix stiffness.

### High RSU-1 mRNA expression is associated with poor prognosis for distant metastasis-free survival (DMFS) and remission–free survival (RFS) in BC patients

**Figure 9:**
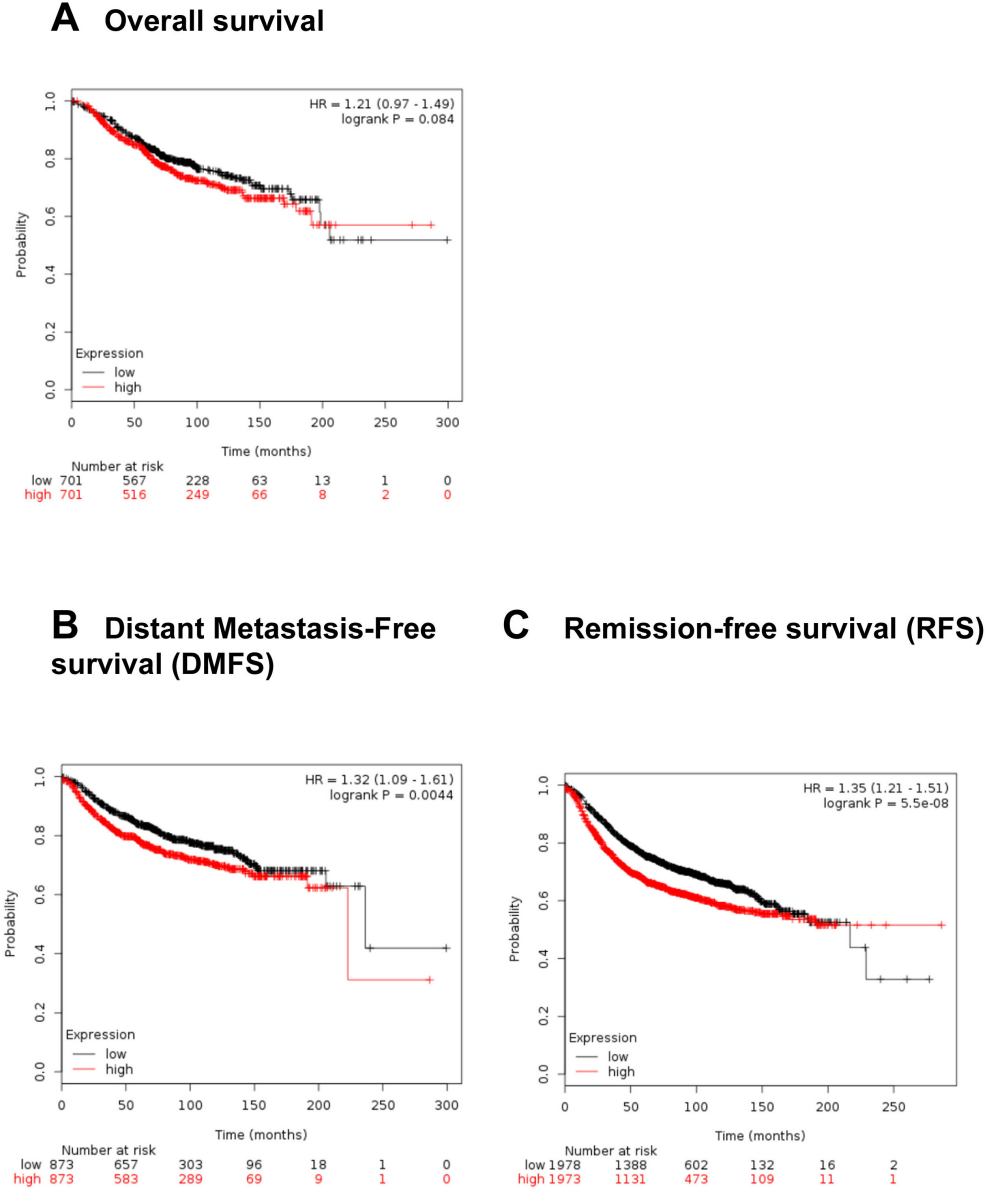
High RSU-1 mRNA expression is correlated with reduced distant metastasis-free survival and reduced remission –free survival in BC patients Kaplan-Meier plotter was used to predict survival of BC patients depending on the level of RSU-1 expression. Overall survival **(A)**, DMFS **(B)** and RFS **(C)** were estimated using the available information on a total of 5,143 BC patients in the database.

To further validate our findings, we utilized the Kaplan Meier plotter, an *in silico* online tool which performs meta-analysis of Affymetrix microarray gene expression data. Meta-analysis involved multiple studies from BC patients [41, 42] to predict survival depending on the level of RSU-1 expression. We first analyzed the data using all the available information on 5,143 BC patients and found that high RSU-1 mRNA expression had no significant effect on overall survival (Figure 9A), but correlated with poor prognosis for distant metastasis-free survival (DMFS) (Figure 9B) as well as remission-free survival (RFS) (Figure 9C).

## DISCUSSION

To better understand the importance of tumor stiffness and cell-ECM interactions in BC progression and metastasis, we recapitulated the actual tumor setting *in vitro* using three BC cell lines of different metastatic potential and two approaches; a) culture of BC cells in 3D collagen gels of increasing stiffness and b) BC cell spheroids, embedded in 3D collagen gels. Our results indicate that the relationship between cells’ invasive capacity and ECM stiffness is biphasic with an optimum invasion in the 1.0 mg/ml collagen gel and the lowest in the 3.0 mg/ml collagen gel, regardless of the aggressiveness of the cells, as it was true for all three cell lines tested (Figure 3). This is in accordance with previous studies showing that cells exhibit biphasic behavior with regard to migration, with an optimum migration velocity in matrices with intermediate-sized pores [43]. Moreover, a recent study by Acerbi et al. showed that there is an optimum range of ECM stiffness for tumor invasion, while ECM remodeling at the tumor invasive front is correlated with increased ECM stiffness [44]. Also, the fact that BC spheroid cell invasion was dramatically reduced in the stiffer matrix condition (Figure 3D-3F) suggests that a smaller pore size in the denser and stiffer 3D matrix may impede formation of cell protrusions, and hence block invasion [45].

Furthermore, we identified RSU-1 as the only one of the cell-ECM adhesion genes examined to respond to the increased stiffness conditions, as its expression was consistently elevated in cells embedded in collagen gels (Figure 4) in all three BC cell lines. In contrast, other cell-ECM adhesion proteins examined, namely ILK, PINCH-1, PARVA, VASP, and Migfilin did not exhibit a consistent pattern of expression in relation to stiffness and/or among the three cell lines (Supplementary Figure 1 and 2). It is worth mentioning however, that gene expression analysis in cells embedded in collagen gels was quite challenging especially at the protein level, and thus collagenase D treatment was selected to facilitate collagen digestion and cell harvesting. Although, it is known that collagenase digestion may interfere with sensitive signaling events such as phosphorylation [46], it did not affect more stable proteins such as cell-ECM adhesion proteins.

Further, RSU-1 was indeed shown to be upregulated in the highly metastatic MDA-MB-231 and MDA-MB-231-LM2 cells compared to the low invasiveness MCF-7 cells (Figure 5), indicating a potential role in aggressiveness. Interestingly, it is clear that RSU-1 is implicated in cell invasion, although its exact role may be cell type specific. More specifically, there are studies showing that RSU-1 promotes cell adhesion, spreading, migration [47], and invasion [19] while there is a study showing that RSU-1 inhibits cancer cell migration and invasion while its alternatively spliced truncated isoform enhances the process [48]. In the present study we show for the first time that RSU-1 silencing leads to reduced UPA, and MMP-13 expression (Figures 6 and 7), both of which are key matrix degradation proteases implicated in cell invasion, and thus metastasis. In fact, although the link between MMP activity, contractility and ECM stiffness has been demonstrated previously [49], RSU-1 has not been implicated in this process to date. Hence, since RSU-1 silencing downregulated UPA, and MMP-13, it is not surprising that RSU-1 depletion also dramatically reduced BC cell spheroid invasion through collagen gels (Figure 8) in all three BC cell lines tested. More importantly, our findings indicate that although RSU-1 is upregulated in increased stiffness conditions (Figure 4), its elimination by siRNA leads to inhibition of spheroid cell invasion regardless of matrix stiffness, suggesting a strong invasion-inhibitory capacity.

Our findings further corroborate recent work showing that RSU-1 depletion from hepatocellular carcinoma cells inhibits cell invasion [19], and additionally demonstrate that this holds true for BC cells as well. It should be noted however, that as RSU-1 has been shown to suppress growth of hepatocellular carcinoma cells [14] and induce apoptosis in breast cancer cells [18], it would be expected that its silencing would promote proliferation and tumor growth and thus perhaps contribute to increased cell migration and invasion. Nevertheless, the fact that RSU-1 depletion leads to such a significant decrease in cancer cell spheroid invasion regardless of collagen concentration or aggressiveness of the BC cell line (Figure 8) indicates that the effect of RSU-1 on invasion is a stronger determinant of the cancer cell phenotype.

Last but not least, analysis of the Kaplan Meier survival plots using the online Kaplan Meier plotter tool corroborated our findings as well as previous work showing RSU-1 correlation with metastasis [18] setting the foundations for its validation as a potential BC metastasis marker.

## MATERIALS AND METHODS

### Antibodies and reagents

Anti-ILK antibody was purchased from Cell Signaling Technology, anti-VASP antibody from Santa Cruz Biotechnology, and anti-β-actin antibody from Sigma-Aldrich. Anti-RSU-1 rabbit polyclonal antibody was kindly provided by Dr. Mary Lou Cutler, Professor at the Uniformed Services University of the Health Sciences, Bethesda USA. Anti-migfilin and anti-PARVA antibodies were a kind gift of Dr. Chuanyue Wu, Professor at the University of Pittsburgh Medical School, Pittsburgh, USA. Collagen I high concentration solution was purchased from Corning, while collagenase D and human insulin solution were obtained from Sigma-Aldrich.

### BC cell lines

BC cell lines MCF-7, and MDA-MB-231 were purchased from ATCC, while MDA-MB-231-LM2 cells were kindly provided by Dr. Joan Massague, Memorial Sloan Kettering Cancer Center [50]. All cells were cultured in Dulbecco's Modified Eagle Medium supplemented with 10% Fetal Bovine Serum, 1% Glutamine and 1% Penicillin/Streptomycin, and incubated in a CO_2_-incubator at 37°C. All cell culture reagents were purchased from Invitrogen.

### Culture of cells in 3D collagen I gels

To increase the stiffness of 3D collagen gels, we increased their concentration [34, 35]. We utilized the high concentration collagen I solution (Corning 354249) and adjusted the pH using a modification of previously published protocols [37]. More specifically, we added the desired amount of collagen I so as to get a final collagen concentration of 0.5, 1.0 or 3.0 mg/ml, in a solution containing 10% 10x Minimal Essential Medium, 1% human insulin solution (Sigma-Aldrich), and distilled water. The pH was adjusted to 7.4 by adding 1N NaOH. Cancer cells were added to the collagen solution before it solidified at a concentration of 2.5 x 10^5^ cells/ml [37] and normal complete culture medium was added on top of the collagen gel containing cells 4h after its solidification. Cells were cultured in 3D collagen gels of 0.5, 1.0 or 3.0 mg/ml for 3 days and were then subjected to gene expression analysis at the mRNA and protein level as specified. In experiments involving RSU-1 silencing, siRNA transfection was performed in traditional 2D culture 1 day prior to embedding cells in the collagen gels. Cells were left to grow in the gels for two more days before being harvested and analyzed for gene expression.

### siRNA transfection

All BC cells were transfected with 100 nM non-specific control siRNA or siRNA against RSU-1 using the HiPerfect reagent (Qiagen). The siRNA used for silencing was purchased from Santa Cruz Biotechnology (sc-90735) whereas the Control siRNA-A (sc-37007) was used as Non Specific Control (NSC) siRNA. Cells were harvested 48h post-transfection and silencing efficiency was verified by western blot and real time PCR as specified in each experiment.

### RNA isolation and real time PCR

Total RNA was extracted from BC cells using Trizol (Invitrogen), purified using RNeasy mini kit (Qiagen) and transcribed to cDNA using Superscript Reverse Transcriptase (Invitrogen). Quantification of gene expression was performed by real-time PCR using CFX96 Real Time PCR (BioRad). B-actin was used as a housekeeping gene. Reactions were done in triplicate and at least 3 independent experiments were performed. All primers used are shown in Supplementary Table 1. Quantification of relative gene expression was performed using the ΔΔCt method. Cells grown in 0.5mg/ml collagen gels and cells transfected with NSC siRNA were used as calibrators, as specified in each experiment.

### Protein extraction and western blot analysis

For protein expression analysis, cells embedded in collagen gels were harvested by scraping and treated with 1mg/ml collagenase D for 30min at 37°C. Cell suspensions were then centrifuged at 300g for 5min to remove collagen and cell pellets were kept for gene expression analysis. Total cell lysates were obtained from cell pellets obtained from 2D culture or collagenase D treatment using 1% sodium dodecyl sulfate in RIPA buffer (20mMTris/Cl pH7.5, 150 mM NaCl, 0.5% NP-40, 1% TX-100, 0.25% sodium deoxycholated, 0.6-2μg/ml aprotinin, 10μM leupeptin, 1μM pepstatin). Protein concentrations in the samples were determined by the BCA protein assay kit (Pierce). Cell lysates were run on a 10-12% acrylamide gel and transferred to a PVDF membrane (Millipore) using the BioRad Semi-dry transfer system (BioRad). Membrane was blocked in 5% non-fat milk in TBST buffer for 1h and was then incubated with appropriate antibodies overnight in 5% milk. Standard western blot procedure steps were followed thereafter using chemiluminescent substrate from Pierce and Kodak Biomax light films. Films were scanned using an HP Scanjet G4010 scanner and images were analyzed using Adobe Photoshop software. Specifically color was discarded and the images were converted to greyscale. No other image manipulation was performed.

### Quantification of protein expression from western blots

RSU-1 (Figure 4C, 5B, 6C and 7E) and PINCH-1 protein expression (Supplementary Figure 2B) were quantified compared to the β-actin loading control using the National Institute of Health Image J software. The mean intensity of respective protein bands from several different immunoblots was used for the quantification, as specified in each figure legend. A p value of 0.05 was considered as statistically significant.

### Cancer cell spheroids formation and spheroid invasion assay

BC cell spheroids were formed using the “hanging drop” technique, as described previously [39, 40, 51]. Briefly, cells were trypsinized, counted and put in suspension at a concentration of 2.5x10^4^ cells/ml. Hanging drops containing 500 cells each were placed on the inside of the cover of a culture dish. Drops were left for at least 24h to allow for spheroid formation. Subsequently, formed spheroids were transferred into wells of a 96-well plate containing 0.5, 1.0 or 3.0 mg/ml collagen gel using a glass Pasteur pipette. Pictures were taken immediately (time zero) using a Nikon Eclipse optical microscope equipped with a digital camera and spheroids were then incubated at 37°C for 5-18h as specified in each experiment and depending on the invasive capacity of each cell type. Cell invasion in surrounding collagen was measured using the Image J software and spheroids’ size (average of the major and minor axis length) at the designated time was compared to the initial size at time zero [30]. In experiments where cells were subjected to RSU-1 siRNA-mediated silencing, siRNA transfection was performed 24h prior to formation of hanging drops and invasion was monitored up until 72h post-transfection. At least 8 spheroids were analyzed per condition and at least three independent experiments were performed.

### Atomic force microscopy

Atomic Force Microscopy (AFM) was used to characterize 3D collagen gels. More specifically, for high resolution imaging of collagen gel fibers AFM images of the collagen films were obtained in air using a Cypher ES™ Environmental AFM microscope (Asylum Research). Briefly, part of the collagen solution (90μl) was flushed on 13mm circular glass cover glasses (AGL46R13, Agar Scientific) and the samples were incubated in a cell culture incubator for 30 min. Samples were then mounted on 15mm specimen AFM metal discs (AGF7003, Agar Scientific). All images were obtained at room temperature in intermittent (also named tapping) mode with AC160TS AFM probes (Olympus). The topographic AFM images are presented in a color scale which represents the Z axis. The surface images were acquired at a fixed resolution (512 × 512 data points) with scan rate between 0.5 and 1 Hz.

For AFM stiffness measurements, an Agilent-Molecular Imaging PicoPlus AFM system (now known as 5500 AFM, Keysight Technologies) was used for force mapping, in contact mode under Phosphate Buffered Saline (PBS). The collagen gel samples were prepared as described in previous section and then were mounted on AFM sample plate and a liquid cell with PBS was used. Force spectroscopy was performed with V-shaped silicon nitride probes (PNP-TR, Nanoword, provided by NanoAndMore). Contact mode images and 8x8 points of force curves were collected and analyzed by AtomicJ [52] so as to calculate the sample's Young's modulus using the Hertz model (for collagen a 0.3 Poisson ratio was used).

The AFM image processing was performed by using the image analysis software that accompanied the AFM, AR SPM Software (ver.14.13.134, Igor Pro 6.37) and the freeware scanning probe microscopy software WSxM 5.0 dev.2.1 [53].

### Kaplan-Meier plotter analysis

Kaplan-Meier plotter, an *in silico* online tool, was used to predict survival of BC patients depending on the level of RSU-1 expression. The Kaplan-Meier plotter uses Affymetrix microarray gene expression data from multiple BC studies and integrates them simultaneously with clinical data including relapse free and overall survival information [41, 42]. Information on a total of 5,143 BC patients was available in the database and we searched for RSU-1 expression in all patients (release 2017, n=5143, all datasets were used for the analysis). Kaplan Meier plotter can be found at: http://kmplot.com/analysis/index.php?p=service&cancer=breast.

### Statistical analysis

Comparison of means using Statgraphics software was used for the statistical analysis. T-test was performed and a *p-*value of <0.05 was considered as statistically significant.

## SUPPLEMENTARY MATERIALS FIGURES AND TABLES



## Abbreviations

AFM: Atomic Force Microscopy
BC: breast cancer
CNV: copy number variation
DMSF: distant metastasis-free survival
ECM: extracellular matrix
ILK: integrin-linked kinase
MMP: metalloproteinases
NSC: non-specific control
LN: lymph node
PARVA: alpha- parvin
PBS: phosphate buffered saline
PINCH-1: Particularly Interesting New C*ysteine-Histidine* rich protein
RFS: remission–free survival
RSU-1: Ras suppressor-1
UPA: Urokinase plasminogen activator
VASP: Vasodilator Stimulated Phosphoprotein
2D: two-dimensions
3D: three-dimensions.
