# Supplementary material for: Identification of Ras suppressor-1 (RSU-1) as a potential breast cancer metastasis biomarker using a three-dimensional in vitro approach
Source: Oncotarget. 2017 Mar 9;8(16):27364–79. doi: 10.18632/oncotarget.16062 (PMC5432341; doi:10.18632/oncotarget.16062)
Supplement: Supplementary file 1 [file oncotarget-08-27364-s001.pdf]

Identification of Ras suppressor-1 (RSU-1) as a potential breast cancer metastasis biomarker using a three-dimensional *in vitro* approach

SUPPLEMENTARY MATERIALS

SUPPLEMENTARY FIGURES AND TABLE

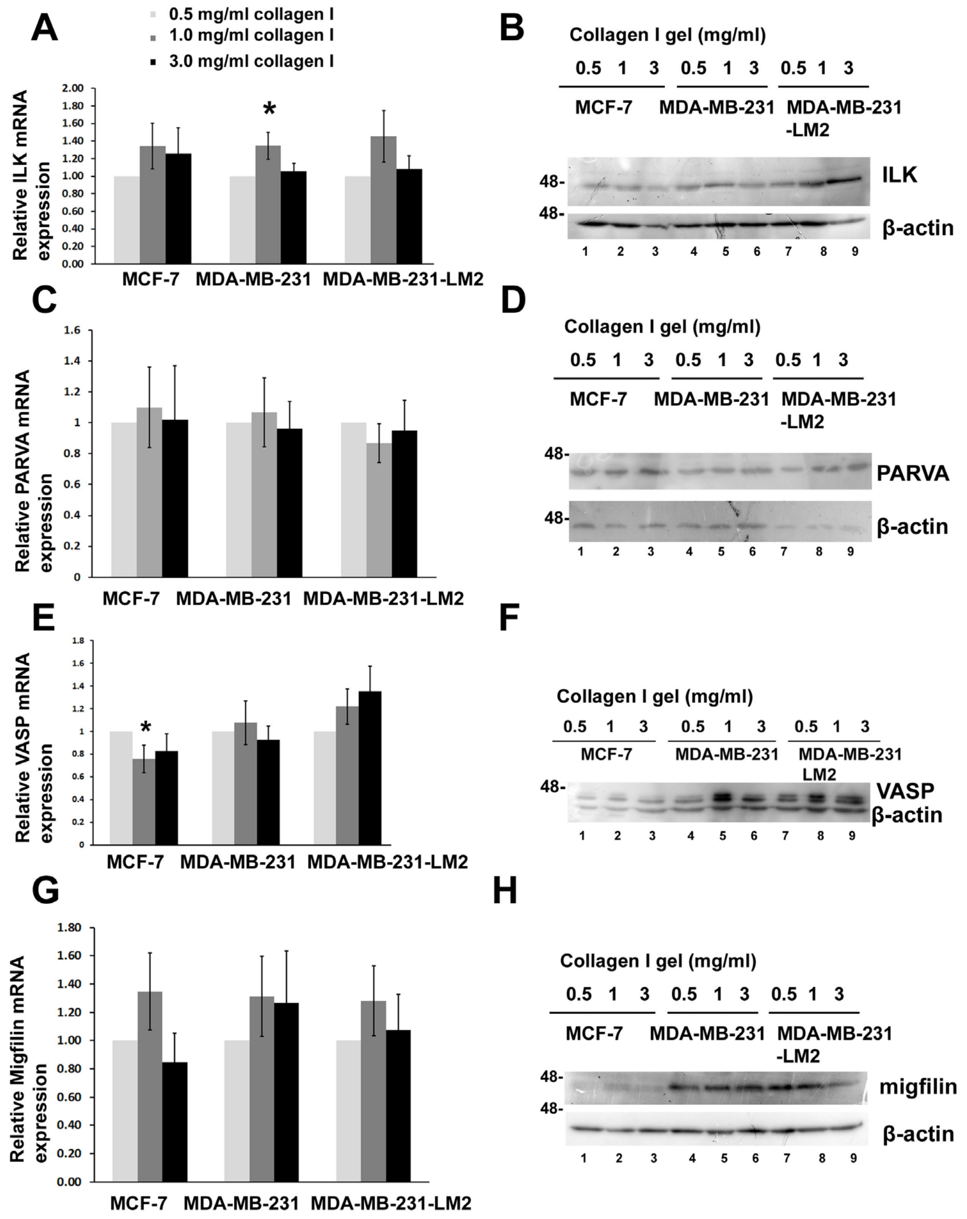

Supplementary Figure 1: No consistent change in ILK, PARVA, Migfilin or VASP in MCF-7, MDA-MB-231 and MDA-MB-231-LM2 cells in relation to increased stiffness. **(A, C, E, G)** Relative mRNA expression of ILK **(A)**, PARVA **(C)**, VASP **(E)** and migfilin **(G)** in MCF-7, MDA-MB-231 and MDA-MB-231-LM2 cells cultured in collagen gels of 0.5, 1.0 and 3.0 mg/ml. At least four independent Real Time PCR experiments were performed, and data were analyzed using the ΔΔCt method and having 0.5mg/ml collagen gel as a calibrator. Asterisks indicate statistically significant changes (p-value<0.05). **(B, D, F, H)** Representative western blots showing protein expression of ILK **(B)**, PARVA **(D)**, VASP **(F)** and migfilin **(H)** in all stiffness conditions in all three BC cell lines. B-actin was utilized as loading control.

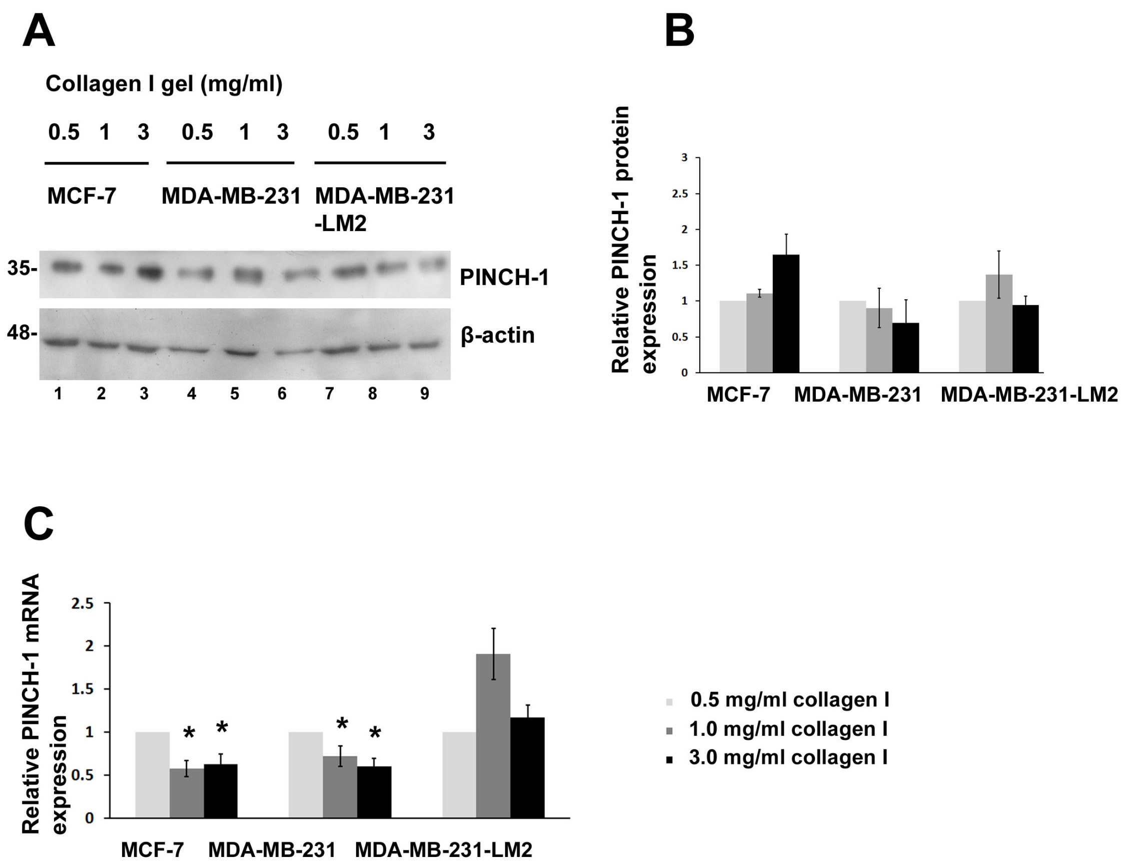

Supplementary Figure 2: No consistent change in PINCH-1 mRNA and protein expression in MCF-7, MDA-MB-231 and MDA-MB-231-LM2 cells in relation to increased stiffness. **(A)** Representative western blot showing PINCH-1 protein expression in all stiffness conditions in all three BC cell lines. B-actin was utilized as loading control. **(B)** Graph representing quantification of PINCH-1 protein expression normalized to the β-actin loading control using NIH Image J software. The mean intensity of PINCH-1 protein bands from 3 different immunoblots was used for the quantification. **(C)** Relative PINCH-1 mRNA expression in MCF-7, MDA-MB-231 and MDA-MB-231-LM2 cells cultured in collagen gels of 0.5, 1.0 and 3.0 mg/ml. Five independent Real Time PCR experiments were performed, and data were analyzed using the ΔΔCt method and having 0.5mg/ml collagen gel as a calibrator. Asterisks indicate statistically significant changes (p-value<0.05).

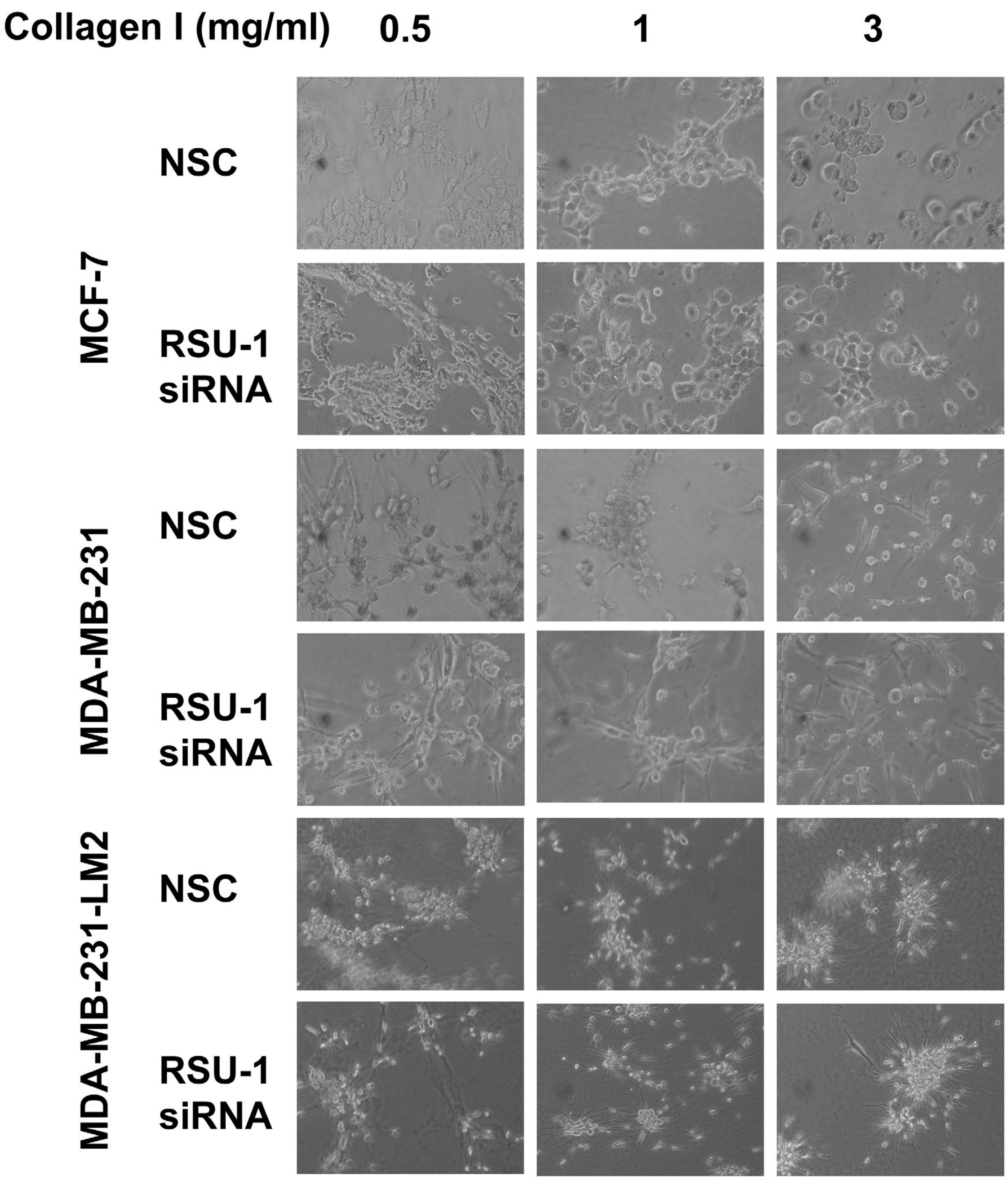

Supplementary Figure 3: Morphology of MCF-7, MDA-MB-231 and MDA-MB-231-LM2 cells following RSU-1 silencing in conditions of increasing matrix stiffness. Morphology of MCF-7 cells grown in 3D culture of 0.5mg/ml, 1.0 mg/ml and 3.0 mg/ml collagen gels after treatment with NSC or RSU-1 siRNA.

Supplementary Table 1: Nucleotide sequence of the primers used for mRNA expression analysis

Primer name	Sequence
ILK	Forward 5′ GAC ATG ACT GCC CGA ATT AG 3′
	Reverse 5′ CTG AGC GTC TGT TTG TGT CT 3′
Migfilin	Forward: 5’CGAATGCATGGGAAGAAACT-3’
	Reverse: 5’GCAGGTTAGGAAGGGAAACC-3’
MMP-13	Forward: 5’TGGCATTGCTGACATCATGA3’
	Reverse: 5’GCCAGAGGGCCCATCAA3’
RSU-1	Forward 5’ AGG CCA CAG AGC AAG GTC TA 3’
	Reverse 5’ CGT GCA ATC TCA AAA GCT CA 3’
PARVA	Forward: 5’-CAATTCGACTCCCAGACCAT-3’
	Reverse: 5’-TGGTCGAACAAGGTGTCAAA-3’
PINCH-1	Forward: 5′ CCG CTG AGA AGA TCG TGA AC 3′
	Reverse: 5′ GGG CAA AGA GCA TCT GAA AG 3′
UPA	Forward: 5’GCTGCTGACCCACAGTGGAA-3’
	Reverse: 5’AAAGTCATGCGGCCTTGGAG-3’
VASP	Forward: 5′GAA AAC CCC CAA GGA TGA AT-3′
	Reverse: 5′GGA AGT GGT CAC CGA AGA AG-3′
β-actin	Forward: 5′-CGAGCACAGAGCCTCGCCTTTGCC-3’
	Reverse: 5′-TGTCGACGACGAGCGCGGCGATAT-3’
